# On the Effect of the Temperature-Humidity Index on Buffalo Bulk Milk Composition and Coagulation Traits

**DOI:** 10.3389/fvets.2020.577758

**Published:** 2020-10-19

**Authors:** Angela Costa, Massimo De Marchi, Sabrina Battisti, Marcella Guarducci, Simonetta Amatiste, Giuseppe Bitonti, Antonio Borghese, Carlo Boselli

**Affiliations:** ^1^Department of Agronomy, Food, Natural Resources, Animals, and Environment, University of Padova, Legnaro, Italy; ^2^Experimental Zooprophylactic Institute of Lazio and Toscana “Mariano Aleandri,” Rome, Italy; ^3^International Buffalo Federation, Rome, Italy

**Keywords:** THI, dairy buffalo, milk coagulation, milk quality, milk acidity, heat stress

## Abstract

Little is known about the effects of high levels of environmental temperature and humidity on milk yield and quality in buffaloes since this species is known to be more heat tolerant than cattle. However, the distribution of sweat glands and the dark skin color can negatively affect heat tolerance. Moreover, due to increased global temperatures, concerns regarding heat stress and thermoregulation in dairy animals, including buffaloes, have been extended to the northern hemisphere. In this study, the effects of both the temperature-humidity index (THI) and the maximum daily temperature-humidity index (MTHI) were estimated on bulk milk traits, namely fat, protein, lactose, urea content, pH levels, somatic cell score, coagulation properties, and bacteria count. The dataset consisted of repeated data from 99 Mediterranean water buffalo farms, and mixed models were used for the analyses. Supporting the negative correlations observed, bulk milk fat, protein, and lactose content were significantly lower when THI and MTHI were higher. Similarly, milk pH was lower when THI and MTHI were high; however, high levels of THI or MTHI seemed to not be markedly associated with the milk's coagulation ability. According to both analysis of variance and correlations, the somatic cell score was not significantly affected by the THI and MTHI. This is the first study based on a large dataset that evaluates the impact of high temperature and humidity in Italian buffalo milk and that provides correlations with traits of interest for the dairy industry, i.e., milk acidity and coagulation ability. In general, findings show that the effects of elevated THI and heat stress on bulk milk quality in buffalo is less evident than in cattle. These preliminary results intend to open debate on the issue of heat stress in dairy buffaloes that are reared in temperate regions. Further studies should focus on individual milk and performance and should investigate the relationship between high THI and buffalo fertility, behavior, and welfare.

## Introduction

The effects of environmental temperature and humidity on milk-related performance, fertility, and welfare have been widely studied in dairy cattle (1–3). In particular, often these two environmental parameters are combined into a unique index, the temperature-humidity index (THI), to evaluate cow heat stress and milk losses, and to monitor the thermoregulation (3). Several formulas have been proposed to calculate THI in dairy cows (4), with the Kibler formula being denoted as the most popular one due to its simplicity and efficacy.

Little is known about the effects of high levels of temperature and humidity on milk yield and quality in buffalo since this species is known to be more heat tolerant than cattle. However, it is worth highlighting that the distribution of sweat glands and the dark color of the skin negatively affect heat tolerance in these animals (5).

Due to increased global temperatures, concerns regarding heat stress and thermoregulation in dairy animals, including buffaloes, has been extended to the northern hemisphere. Italy hosts the majority of European buffaloes (6); the water buffalo has been traditionally present and farmed in the southern regions of the country, particularly where Mozzarella di Bufala Campana cheese, which has been granted a “protected designation of origin” status, is manufactured (7).

To limit the negative impact of high THI during the summer and improve animal welfare, showers and foggers are installed in some Italian farms to reduce environmental temperatures and decrease both the respiratory rate and body temperature of buffaloes. Despite this, cheese industries still consider the summer milk to be the worst for cheesemaking.

Thus, in the present study, the effects of different levels of THI and maximum temperature-humidity index (MTHI) on milk quality traits have been estimated using data of bulk milk samples collected during the year 2018.

## Materials and Methods

### Herds

Data collection was carried out in 99 commercial Mediterranean water buffalo herds located in the Latium region (Italy), in the area of Mozzarella di Bufala Campana cheese. Bulk milk sampling lasted 12 months, from January 2018 to December 2018. The geographical coordinates of each herd were obtained from the national database of the zootechnical registry established by the Ministry of Health at the National Service Center of the Experimental Zooprophylactic Institute of Abruzzo and Molise (Teramo, Italy). At least three milk samples were available for each herd.

### Temperature and Humidity Index

Climatic data consisted of daily average and maximum temperatures (°C) and relative humidity, and were extrapolated from the Lazio Region Integrated Agrometeorological Service database (SIARL; http://www.arsial.it/portalearsial/Agrometeo/E7.asp). Data were recorded by 26 meteorological stations with known coordinates (latitude and longitude) and distributed between the provinces of Frosinone and Latina (Latium region), i.e., where the herds were located. Through the geographical coordinates of weather stations and herds, a spatial interpolation was performed by the “inverse distance weighted” technique using the ArcGis 10.3.1 software in order to identify the closest weather station for each herd. In ArcGis 10.3.1 software, the “extract multi values to points” technique allowed for the creation of a database where each herd in each sampling date had unique values of the daily average (AT) and maximum temperature (MT) and relative humidity (RH).

According to the literature (4, 8, 9), THI and MTHI were calculated using Kibler's equations:

$$\begin{array}{l} THI=\left(1.8\;\cdot \;AT\right)-\left(1.0-\;RH\right)\cdot \left(AT-\;14.3\right)+32\;;\\ MTHI=\left(1.8\;\cdot \;MT\right)-\left(1.0-\;RH\right)\cdot \left(MT-\;14.3\right)+32\;. \end{array}$$

In particular, the MTHI represents the worst daily environmental conditions experienced by the lactating buffaloes and was calculated based on suggestions found in the literature (9); in fact, sensitivity of test-day milk yield to MTHI was found to be greater compared with THI in cattle.

### Milk Quality Determination

Milk samples were refrigerated (4°C) and analyzed within 24 to 36 h from collection at the Experimental Zooprophylactic Institute of Lazio and Tuscany (Rome, Italy), the national reference laboratory for dairy product quality in Central Italy. Content (%) of fat (FC), protein (PC), lactose (LC), milk urea nitrogen (MUN, mg/dL), pH, and electrical conductivity (EC, mS) were determined by the MilkoScan FT6000 (FOSS Electric, Hillerød, Denmark), calibrated with appropriate buffalo standards. Somatic cell count (SCC, cells/mL) and total bacterial count (TBC, CFU/mL) were recorded by Fossomatic and Bactoscan, respectively (FOSS Electric, Hillerød, Denmark). To achieve a normal distribution of the SCC data, the somatic cell score (SCS, units) was calculated as: SCS = log_2_ (SCC/100,000) + 3 (3). For the same reason, all values of TBC were log-transformed to LTBC, as LTBC = log_10_ (TBC). The limit fixed by the EU Regulation no. 853 of 2004 for buffalo milk TBC is 1,500,000 CFU/mL; however, this limit is reduced to 500,000 CFU/mL if the milk used to manufacture dairy products is not heat treated (10).

Milk clotting parameters were determined through reference method by the Formagraph (FOSS Electric, Hillerød, Denmark) using animal rennet according to conventional protocols (11, 12). Briefly, each milk sample (10 mL) was heated to 35°C and added with 200 μL of commercial calf rennet (75% chymosin and 25% bovine pepsin, Caglificio Clerici SPA-Sacco SRL, Cadorago, Italy) diluted (1:1) in distilled water. The milk clotting parameters recorded were:

- RCT (rennet coagulation time, min): interval between the rennet addition and beginning of coagulation;
- k_20_ (curd firming time, min): the time necessary to obtain a curd firmness of 20 mm; and
- a_30_ (curd firmness, mm): the curd firmness recorded after 30 min of rennet addition.

### Statistical Analysis

**Figure 1:**
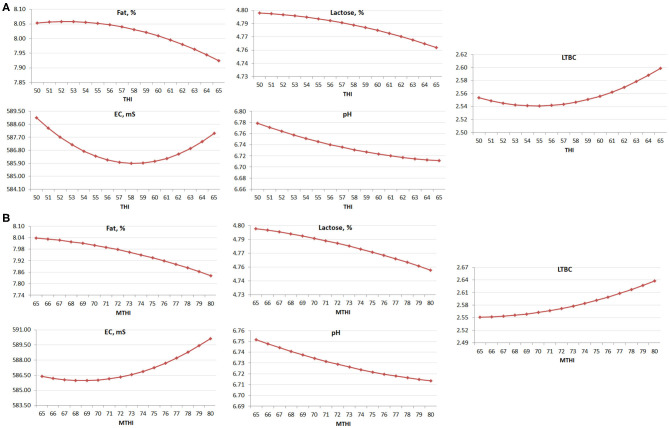
Regression (linear + quadratic) of **(A)** THI and **(B)** MTHI on the studied milk traits (*P* < 0.05).

Regression coefficients were obtained with a mixed model in SAS software v. 9.4 (SAS Institute Inc., Cary, NC), where THI (or MTHI) was included as linear and quadratic covariate (fixed effects) and where the farm was the random factor. The regressions were performed in order to test the significance of the effect for each milk feature (Figure 1). For the analysis of variance, the mean, and the standard deviation (SD) of THI (Table 1) were used to design three classes, as follows:

- Class 1 (low): if THI < mean – 1 SD;
- Class 2: if THI ≥ mean – 1 SD and < mean + 1 SD; and
- Class 3 (high): if THI ≥ mean + 1 SD.

**Table 1:** Descriptive statistics of temperature-humidity index (THI), maximum of temperature-humidity index (MTHI), and milk traits in the whole dataset.

**Trait**	***n***	**Mean**	**Standard deviation**	**Min**.	**Max**.
THI	1,390	58.44	10.50	39.66	76.09
MTHI	1,390	68.66	12.59	45.65	91.65
Milk traits					
Fat, %	1,385	7.95	0.66	5.93	9.95
Protein, %	1,381	4.49	0.24	3.72	5.25
Lactose, %	1,374	4.78	0.13	4.35	5.22
Milk urea nitrogen, mg/dL	1,386	34.00	9.24	8.20	61.90
Electrical conductivity, mS	1,379	589.27	27.70	513.20	684.40
Rennet coagulation time, min	1,357	13.25	4.53	2.45	27.30
Curd firmness, mm	1,318	49.28	10.09	11.22	88.00
Curd firming time, min	1,323	3.96	2.63	0.20	16.30
pH	1,383	6.75	0.10	6.46	7.06
Somatic cell score	1,390	3.52	0.81	1.11	6.30
Log_10_ of total bacteria count	1,353	2.47	0.66	0.60	3.70

At the end, classes 1, 2, and 3 of THI included 24.25, 51.58, and 24.17 % of the observations, respectively.

The following model was used to estimate the effect of the level of THI on milk quality traits in the SAS software v. 9.4 (SAS Institute Inc., Cary, NC)

$$\begin{array}{l} y_{ijk}=\mu +Thi_{i}+Herd_{j}+e_{ijk}, \end{array}$$

where *y*_*ijk*_ is the bulk milk trait investigated (FC, PC, LC, MUN, EC, pH, SCS, LTBC, and the clotting parameters); μ is the overall intercept of the model; *Thi*_*i*_is the fixed effect of the ith class of THI (j = 1 to 3); *Herd*_*j*_ is the random effect of the jth herd (≥3 at least 3 bulk milk records per herd); and *e*_*ijk*_ is the random residual. A multiple comparison of least squares means (LSM) for the fixed effects was performed using the Bonferroni's test (*P* < 0.05).

The same approach was used to estimate the effect of the level of MTHI on the same milk quality traits. In particular, MTHI was a fixed effect with three classes, as follows:

- Class 1 (low): if MTHI < mean – 1 SD;
- Class 2: if MTHI ≥ mean – 1 SD and < mean + 1 SD; and
- Class 3 (high): if MTHI ≥ mean + 1 SD.

The mean and SD of MTHI are given in Table 1 and the frequency of observation in classes 1, 2, and 3 was 26.91, 53.45, and 19.64%, respectively.

## Results

**Figure 2:**
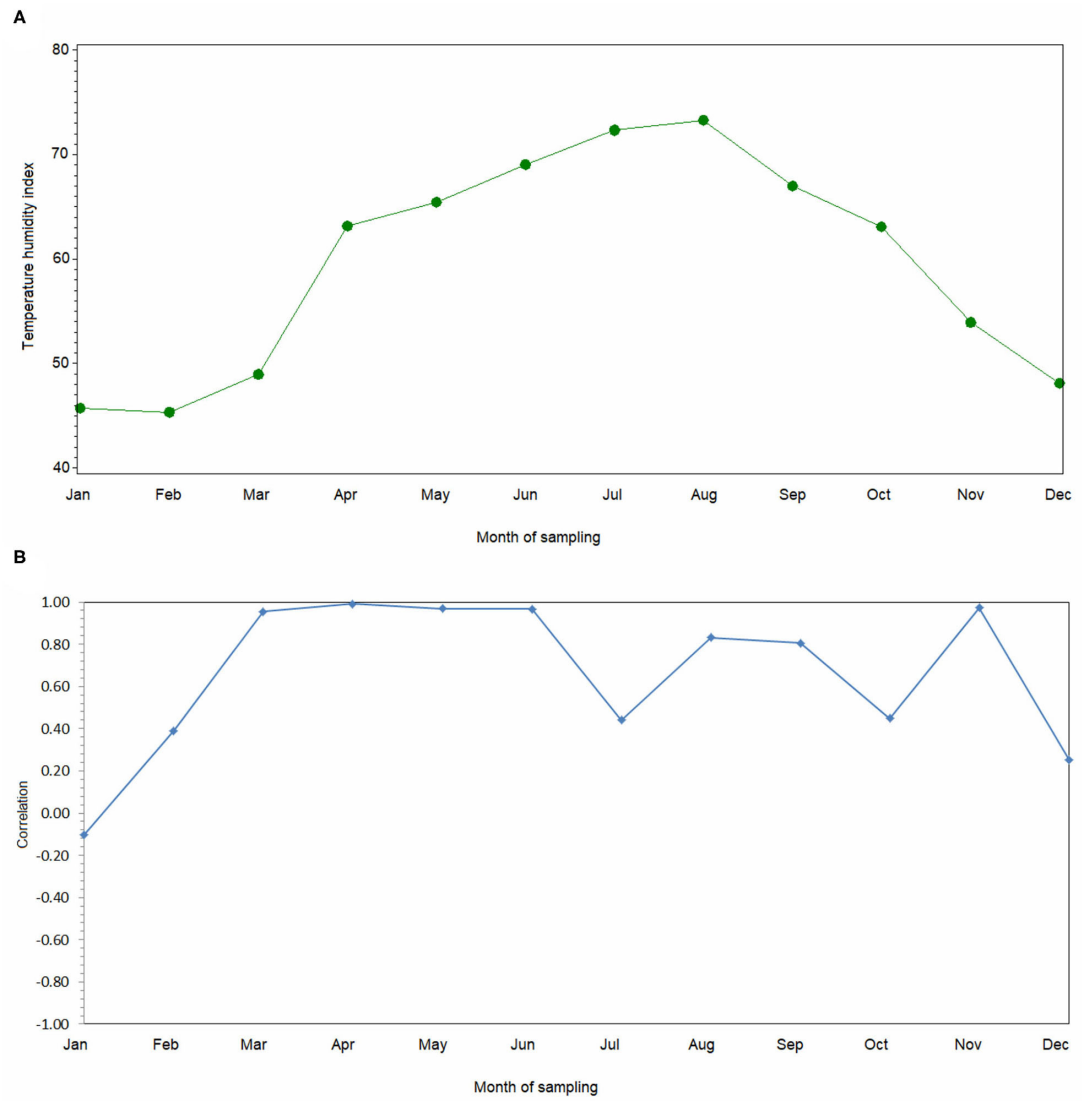
Trend of **(A)** average temperature-humidity index and **(B)** correlation between temperature-humidity index and maximum temperature-humidity index during 2018.

Overall, the THI reached a maximum in August and a minimum in January (Figure 2A) and, on average, the THI was 58.44 while the MTHI was 68.66 (Table 1). The greatest MTHI value recorded by the stations was 91.65 (Table 1). The correlation between THI and MTHI was overall equal to 0.98 (*P* < 0.001, data not shown), but their association showed variation across the months (Figure 2B). In particular, the two indices were less correlated in winter.

Descriptive statistics of milk composition traits are depicted in Table 1; according to the mean and SD of each trait, the most variable feature was k_20_ with a coefficient of variation of 66.4%, followed by RCT, MUN, and LTBC. The trait with the lowest coefficient of variation (2.7%) was LC, followed by EC and PC.

Milk quality features and coagulation traits were overall weakly to moderately correlated with each other and some associations were not significant (Table 2). In particular, the strongest correlations were those between FC and PC (0.54), RCT and a_30_ (−0.48), pH and FC (−0.46), pH and LC (0.45), and LC and EC (−0.33).

**Table 2:** Pearson correlations (*P* < 0.05) between bulk milk fat (%, FC), protein (%, PC), lactose (%, LC), urea nitrogen content (mg/dL, MUN), electrical conductivity (mS, EC), rennet coagulation time (min, RCT), curd firmness (mm, a_30_), curd firming time (min, k_20_), pH, somatic cell score (SCS), and log_10_ of total bacteria count (LTBC).

**Trait**	**FC**	**PC**	**LC**	**MUN**	**EC**	**RCT**	**a_**30**_**	**k_**20**_**	**pH**	**SCS**
PC	0.54									
LC	−0.21	−0.33								
MUN	ns	−0.07	−0.09							
EC	−0.33	ns	−0.36	−0.12						
RCT	0.09	0.16	ns	ns	−0.07					
a_30_	0.11	0.08	−0.06	ns	−0.05^a^	−0.48				
k_20_	ns	ns	ns	ns	ns	ns	−0.44			
pH	−0.46	−0.26	0.45	−0.31	0.09	0.07	−0.12	ns		
SCS	0.06	0.19	−0.19	ns	0.24	0.06	−0.06	0.05^a^	−0.06	
LTBC	−0.13	−0.16	−0.10	ns	0.17	−0.22	ns	ns	−0.12	0.06

a *0.05 ≤ P < 0.10*.

**Figure 3:**
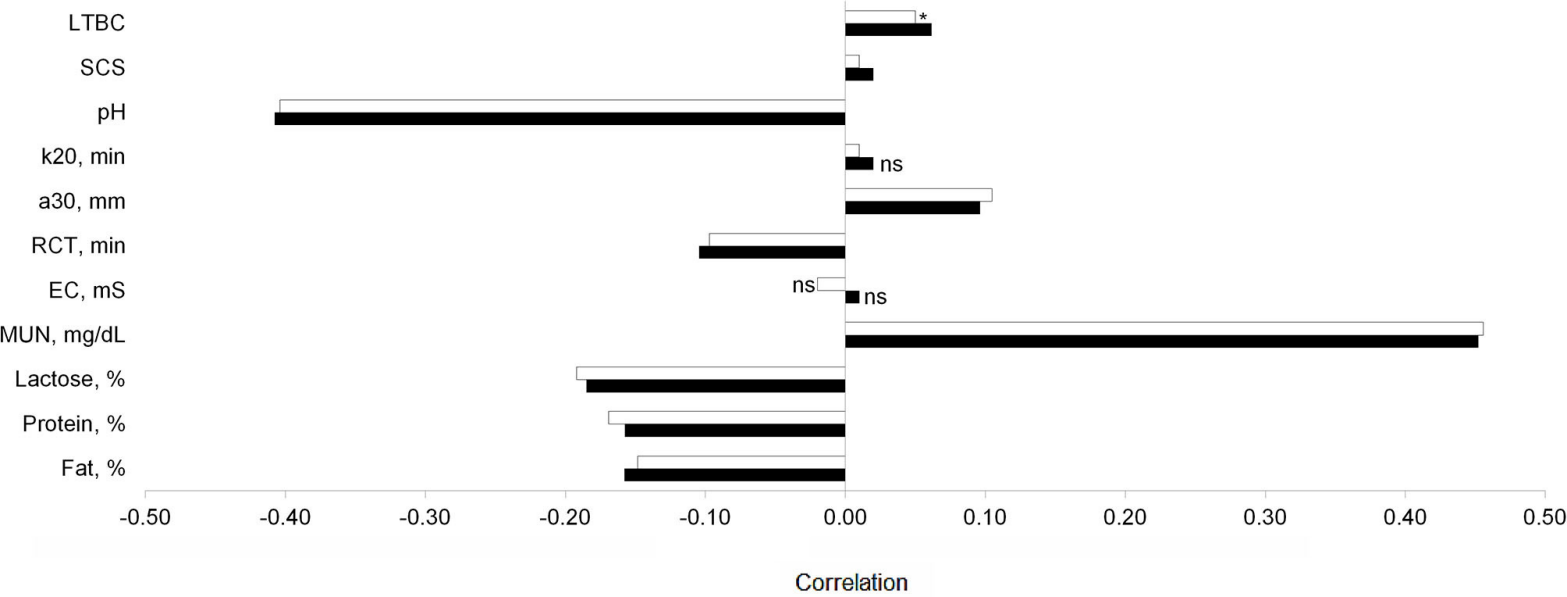
Pearson correlations (*P* < 0.05) of temperature-humidity index (black bar) and maximum temperature-humidity index (white bar) with milk composition traits, urea nitrogen (MUN), electrical conductivity (EC), rennet coagulation time (RCT), curd firmness (a_30_), curd firming time (k_20_), somatic cell score (SCS), and log_10_ of total bacteria count (LTBC). Asterisk indicates 0.05 ≤ *P* < 0.10.

The correlations of THI with milk traits were similar to those of MTHI (Figure 3); in fact, the strongest correlations of both THI and MTHI were with MUN and pH (Figure 3). Both THI and MTHI were also significantly positively correlated with LTBC, although weakly.

In general, the associations with major milk solids (FC, PC, and LC) and with RCT were negative with a moderate to weak magnitude. Furthermore, the correlations of THI and MTHI with a_30_ were positive but weak, i.e., <0.15 (Figure 3).

The mean, SD, min, and max for THI and MTHI for each class is presented in Table 3. Except for RCT, k_20_, and SCS all milk traits studied were significantly affected by the class of THI (Table 4). In fact, milk in the high THI class (class 3) was characterized by the lowest solid content, i.e., FC, PC, and LC. On the contrary, the lowest LSM of MUN was observed in class 1 (Table 4). Milk pH was the lowest in class 3, where acidity was thus more favorable for the coagulation.

**Table 3:** Descriptive statistics of temperature-humidity index (THI) and maximum of temperature-humidity index (MTHI) for each class.

**Class**	***n***	**Mean**	**Standard deviation**	**Min**	**Max**
**THI**					
1 (low)	337	45.02	1.36	39.66	47.72
2	717	58.53	6.58	47.98	68.94
3 (high)	336	71.72	209	68.95	76.09
**MTHI**					
1 (low)	374	52.62	2.71	45.65	56.06
2	743	70.64	7.44	56.07	81.24
3 (high)	273	85.25	2.79	81.31	91.65

**Table 4:** Significance of linear (*P*_L_) and quadratic (*P*_Q_) regression coefficient of temperature-humidity index (THI) and least squares means of bulk milk traits for the fixed effect of THI with significance (*P*) of the effect (three classes).

**Trait**	***P*_**L**_**	***P*_**Q**_**	**Class of THI**			
			**1 (low)**	**2**	**3 (high)**	***P***
Fat, %	***	***	8.00^a^	8.00^a^	7.70^b^	***
Protein, %	ns^1^	ns	4.54^a^	4.49^b^	4.36^c^	***
Lactose, %	**	**	4.80^a^	4.79^a^	4.73^b^	***
Milk urea nitrogen, mg/dL	ns	ns	27.81^c^	33.61^b^	39.18^a^	***
Electrical conductivity, mS	***	***	594.36^a^	588.56^ab^	592.67^b^	**
Rennet coagulation time, min	ns	ns	13.42^a^	13.22^a^	12.78^a^	ns
Curd firmness, mm	ns	ns	48.15^b^	49.73^a^	49.40^ab^	*
Curd firming time, min	ns	ns	3.94^a^	3.90^a^	4.10^a^	ns
pH	***	***	6.83^a^	6.74^b^	6.71^c^	***
Somatic cell score	ns	ns	3.53^a^	3.56^a^	3.51^a^	ns
Log_10_ of total bacteria count	**	**	2.61^ab^	2.58^b^	2.68^a^	*

Different letters within row indicate statistical significance (P < 0.05). ***P < 0.001, **P < 0.01, *P < 0.05, ^ns^not significant.

*^1^P_L_ = 0.06*.

The effect of the MTHI was not significant for k_20_ and SCS (Table 5). The worst milk composition in terms of FC, PC, and LC was observed in class 3 (high). In the same class, LTBC and MUN were the highest (Table 5). Finally, milk pH was greater when MTHI was lower, i.e., in class 1.

**Table 5:** Significance of linear (*P*_L_) and quadratic (*P*_Q_) regression coefficient of maximum temperature-humidity index (MTHI) and least squares means of bulk milk traits for the fixed effect of MTHI with significance (*P*) of the effect (three classes).

**Trait**	***P*_**L**_**	***P*_**Q**_**	**Class of MTHI**			
			**1 (low)**	**2**	**3 (high)**	***P***
Fat, %	***	***	8.01^a^	7.97^a^	7.66^b^	***
Protein, %	ns^1^	ns	4.56^a^	4.47^b^	4.44^c^	***
Lactose, %	**	**	4.80^a^	4.79^b^	4.73^c^	***
Milk urea nitrogen, mg/dL	**	ns	27.70^c^	34.37^b^	39.38^a^	***
Electrical conductivity, mS	***	***	595.36^a^	587.98^b^	593.65^a^	***
Rennet coagulation time, min	ns	ns	13.83^a^	12.97^b^	12.78^b^	**
Curd firmness, mm	ns	ns	46.98^b^	50.10^a^	49.80^a^	***
Curd firming time, min	ns	ns	3.96^a^	3.92^a^	4.06^a^	ns
pH	***	***	6.82^a^	6.73^b^	6.72^b^	***
Somatic cell score	ns	ns	3.53^a^	3.56^a^	3.49^a^	ns
Log_10_ of total bacteria count	**	**	2.59^a^	2.60^a^	2.69^b^	*

Different letters within row indicate statistical significance (P < 0.05). ***P < 0.001, **P < 0.01, *P < 0.05, ^ns^not significant.

*^1^P_L_ = 0.07*.

Considering the regression coefficients (linear and quadratic) of THI a significant effect was observed for some traits (Figure 1A), with the exception of PC, MUN, coagulation properties, and SCS (Table 4). Despite this, the linear effect of THI on PC was almost significant, with *P* = 0.06. The linear and the quadratic effects of MTHI (Figure 1B) were not significant for PC, coagulation properties and SCS and, in the case of MUN, only the linear effect was significant (Table 5). Despite this, the linear effect of MTHI on PC was close to the significance threshold, with *P* = 0.07.

## Discussion

Descriptive statistics observed in this study for FC, PC, LC, SCS, and coagulation traits mirrored those reported in studies based on bulk milk data collected on 36 Italian buffalo herds (11) and on 1,414,449 individual buffalo milk test-day records (7). As expected, the variability of the bulk milk traits was lower compared with individual samples (7). However, a comprehensive comparison with the literature was not possible, since buffalo bulk milk data collected on a large scale are usually scarcely available.

Pearson correlations (Table 2) were overall in agreement with those reported in literature for Mediterranean water buffalo and based on individual milk samples (7, 13). Moreover, some correlations, like those of THI with milk FC, PC, and LC, were in line with the trend observed for the LSM in the three classes of THI (Table 4). Milk samples collected in presence of high THI or high MTHI (class 3) showed more favorable coagulation properties but a lower concentration of solids compared with class 1 (Tables 4, 5). To our knowledge only few studies have investigated the effects of THI on milk quality traits in buffalo worldwide and, overall, FC usually is affected by THI. In particular, a reduction of about 13% in milk FC has been observed between the cold and hot season (14) in individual milk. In this study, a considerable decrease of FC was observed moving from class 1 and 2 to class 3 (Tables 4, 5). In the case of Egyptian and Murrah buffaloes (14, 15), authors have reported a significant reduction of milk FC and of all solids content with increasing environmental temperature. The relationship between THI and milk quality traits has been recently investigated in 51 bulk milk samples (16); the results confirmed those of the present study, evidencing the greatest FC (8.57%) in January and the lowest (6.50%) in the summer season. On the opposite, PC was not significantly affected by the season of sampling (16, 17).

Concerning SCS and LTBC, the effect of THI and MTHI was not clear; this was also confirmed by the weak correlations (Figure 3). In the same area, the variation of SCC (average of 137,692 cells/mL) and bacteria count (average of 251,013 UFC/mL) across months was investigated (18); authors found not-significant variation for SCC, while the bacteria count increased during the warm months.

Both THI and MTHI significantly affected MUN, with more favorable (lower) LSM in class 1 (Tables 4, 5). These results were consistent with a study investigating the variation of serum and milk urea in the presence of heat stress in buffaloes (19). In particular, the serum urea concentration recorded in July was significantly greater than the one recorded in May, likely because of an increase in gluconeogenesis, the catabolism of amino acids and rumen ammonia levels during hot and humid periods.

In regard to coagulation traits, some correlations and LSM apparently seem to suggest a favorable association between a_30_ and THI and between RCT and MTHI; however, it is important to highlight that from a cheese making point of view, shorter RCT and greater a_30_ are desirable, but in some cases this situation corresponds to a compromise between milk acidity and microbiological profile. Milk k_20_ was not affected by either THI or MTHI.

## Conclusions

In this study the effects of THI and MTHI were estimated on bulk milk quality traits in Mediterranean water buffalo. This is the first study using a large dataset to evaluate the impact of high environmental temperature and humidity in this species in Italy and to provide correlations of traits of interest for the dairy industry, including clotting parameters. In general, the findings show that the effects of heat stress on buffalo milk quality traits are less evident than in cattle and, therefore, comparisons and parallelisms with studies dealing with the effects of THI in bovines may be not appropriate. Milk solids content was impaired by high THI and MTHI; in fact, both FC and PP, which are important for cheese making, were significantly lower when the temperature and humidity were higher, and this was supported by the negative correlations observed.

Milk pH was lower and more favorable when THI and MTHI were high, however, on the other hand, the impact of high levels of THI or MTHI on the three coagulation properties was not clear. Specific strategies based on precision livestock farming techniques should be studied to reduce the impact of heat stress in the Italian buffalo.

This study should be therefore considered preliminary, with the purpose of opening the debate in the scientific community on the issue of heat stress in dairy buffaloes that are reared in the northern hemisphere. Further studies should focus on individual milk and explore the relationship between high THI and animal reproductive performance, behavior, and welfare.

## Data Availability Statement

The raw data supporting the conclusions of this article will be made available by the authors, without undue reservation.

## Ethics Statement

Ethical review and approval was not required for the study because milk data routinely available were used and thus no interaction with animals was present. Written informed consent was obtained from the owners for the participation of their animals in this study.

## Author Contributions

The work was conceived and designed by CB, AB, and MD. SB, MG, SA, and GB were in charge to set up the experimental design and to follow milk sampling. AC and CB performed the analyses and interpreted the results. The manuscript was mainly written by AC, CB, MD, and AB. All authors have approved the final version.

## Conflict of Interest

The authors declare that the research was conducted in the absence of any commercial or financial relationships that could be construed as a potential conflict of interest.
